# Informatics Metrics and Measures for a Smart Public Health Systems Approach: Information Science Perspective

**DOI:** 10.1155/2017/1452415

**Published:** 2017-01-10

**Authors:** Timothy Jay Carney, Christopher Michael Shea

**Affiliations:** ^1^The Gillings School of Global Public Health, The University of North Carolina at Chapel Hill, 1101-C McGavran-Greenberg Bldg., CB 7411, Chapel Hill, NC 27599-7411, USA; ^2^The Gillings School of Global Public Health, The University of North Carolina at Chapel Hill, 1101-F McGavran-Greenberg Bldg., CB # 7411, Chapel Hill, NC 27599-7411, USA

## Abstract

Public health informatics is an evolving domain in which practices constantly change to meet the demands of a highly complex public health and healthcare delivery system. Given the emergence of various concepts, such as learning health systems, smart health systems, and adaptive complex health systems, health informatics professionals would benefit from a common set of measures and capabilities to inform our modeling, measuring, and managing of health system “smartness.” Here, we introduce the concepts of organizational complexity, problem/issue complexity, and situational awareness as three codependent drivers of smart public health systems characteristics. We also propose seven smart public health systems measures and capabilities that are important in a public health informatics professional's toolkit.

## 1. Introduction 

Public health informatics is an evolving domain in which practices constantly change to meet the demands of a highly complex public health and healthcare delivery system. The typical definition for a variety of domains of informatics (e.g., public health, population health, nursing, clinical, medical, health, consumer, and biomedical) centers on the “application of information science and information technology to [a specific domain of] practice, research, and training” [1, 2]. This definition of informatics relies on a technical view of the health system. A technical view of informatics largely identifies more tangible products such as databases, decision-support tools, information systems, web portals, and mobile devices as the primary means of addressing complex health issues, improving care, and reducing health disparities.

**Figure 1:**
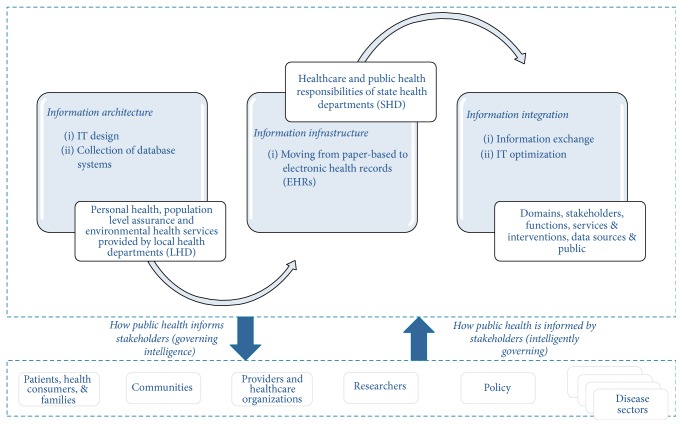
Public health informatics systems intelligence perspectives.

Public health informatics systems expressed as a function of intelligence can be understood in terms of two codependent pathways of (1) generating health information technology (HIT) policies that ensure our ability to* Govern Intelligence* as a byproduct and (2) allowing innovations in HIT to shape and inform public health systems policy and practice to ensure that we* Govern Intelligently*. In the former case, public health informatics professionals endeavor to generate HIT policy to guide national, state, and local information architecture, information infrastructure, and information integration efforts that ultimately guide how public health meets the needs of stakeholder/agents such as patients/families/health consumers, communities, providers/healthcare organizations, researchers, policymakers, and disease-centric communities of practice through the meaningful supply of intelligence. Such intelligence can inform stakeholder understanding about the burden of disease, spread of an outbreak, health alerts and food recalls, disease clusters, community needs assessments, and health risk assessments. In the latter case, public health informatics professionals seek to find innovative ways to leverage HIT to improve the way we govern by seeking ways to streamline processes that positively impact cost, quality, safety, and overall health outcomes.  Figure 1 highlights these relationships in the context of public health practice domains.

Although useful for fostering greater levels of adoption and use of technical measures, this technical view of public health informatics (1) does not highlight the changing knowledge needs of these system agents over time, (2) fails to capture the full array of interaction among agents in a dynamic environment, and (3) cannot maintain pace in adapting to an ever-increasing complex environment. In other words, the purely technical approach does not effectively highlight the full spectrum of knowledge, communication, and learning that is needed to keep all types of health system stakeholders—including individuals, organizations, or collections of individual and organizational networks (e.g., coalitions, collaborations, consortiums, and taskforces)—informed and able to respond to environmental changes at all stages of the healthcare continuum.

In this era of informatics where the emphasis is on less tangible cognitive capacities (e.g., learning health systems, intelligent and smart systems, and complex adaptive systems), a new public health informatics analytics approach may be required that is less information technology-driven and more knowledge-driven and defines new ways of demonstrating the added value of informatics in shaping health systems performance [3]. Specifically, stakeholders (hereafter referred to as individual- or organizational-level agents) need concise, accurate, and objective analytic measurements of abstract concepts, such as empowerment, which previously has been described as a function of knowledge for the purposes of achieving a quantifiable metric for computational analysis of performance.

Such a view of public health informatics may focus on abstract constructs like actionable intelligence as the primary informatics-centric outcome [3]. Such a strategy should yield objective operational measures and capabilities designed to ensure that individual agents, organizations, and networks have sufficient knowledge to mount an intelligent response to solve complex public health problems. In other words, the strategy should support development and maintenance of smart health systems, that is, a system that “incorporates functions of sensing, actuation, and control in order to describe and analyze a situation, and make decisions based on the available data in a predictive or adaptive manner, thereby performing smart actions. In most cases the ‘smartness' of the system can be attributed to autonomous operation based on closed loop control, energy efficiency, and networking capabilities” [4].

The purpose of this paper is to propose a set of measures for tracking the development and sustainability of smart public health systems. Specifically, we introduce the concepts of organizational complexity, problem/issue complexity, and situational awareness as three codependent drivers of smart health systems. We then describe seven smart health systems measures. This discussion is important for public health informatics professionals responsible for specifying metrics, overseeing information systems housing data for the metrics, and evaluating the performance of smart public health systems.

## 2. Factors Shaping Smart Agents and Organizations 

The underlying objective of any agent or actor within a given public health system is to maximize the use of data, information, and knowledge as strategic resources. An informatics-biased view of a public health system focuses on the sum of data, information, knowledge systems, people, practices, policies, and cultural factors that operates to support some predefined intelligence strategy, organizational mission, or other event [5]. In these terms, the public health system can be understood as a functional knowledge culture. We have also used related terms such as knowledge environments, information or knowledge ecosystems, and information or knowledge ecologies to represent this idea. Here, we use knowledge culture and knowledge environment interchangeably. We argue that any defined system boundary that contains the formal or informal governance of critical strategic and shared knowledge resources can be called a knowledge environment. The primary purpose of any knowledge environment is best understood in terms of the essential need to leverage data, information, and knowledge in managing individual or collective uncertainty [6–8].

**Figure 2:**
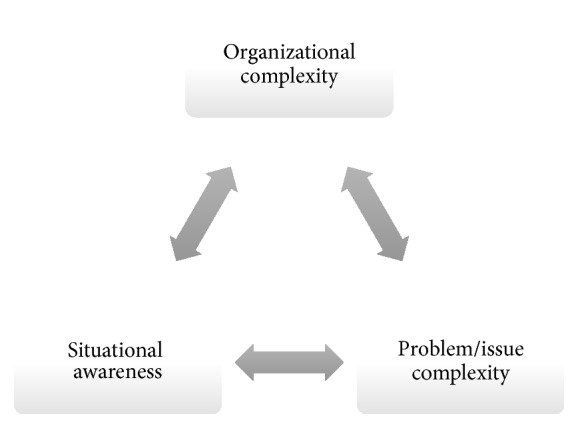
Knowledge environment factors of influence.

The way in which we engage in information and knowledge seeking, organize ourselves into collectives of varying unit configurations (e.g., workgroups, project teams, taskforces, departments, divisions, networks of organizational coalitions, and consortiums), and/or apply the use of tools or technology indicates the basic need to manage any and all forms of uncertainty [9]. We organize ourselves in response to external and internal drivers/stressors and increasing environmental complexity as a means of reducing or removing any impediments toward fast, reliable, and pertinent data, information, and knowledge resources [10]. This imperative to organize for the sake of becoming smarter is best observed in our introduction of three primary drivers that we argue are interdependent in any knowledge environment. By describing these factors as interdependent, we are stating that as one type of driver category increases or decreases by some set of circumstances or events, corresponding changes can occur in one or both of the other areas. These areas include organizational complexity, problem/issue complexity, and situational awareness (see Figure 2). Essentially, each of these three primary driver categories shapes our overall data, information, and knowledge strategy within any knowledge environment. The primary objective of an informatician in designing and maintaining a smart public health knowledge environment is then to understand the basic predictors of change in any or all of these categories, as well as to account for the corresponding mediation/moderation factors that can shape continued data, information, and knowledge maximization for agents within any public health knowledge environment.

### 2.1. Organizational Complexity Factors Shaping Public Health Knowledge Environments

We use a variety of organizational structures to facilitate interaction, communication, and knowledge representation in our quest to manage changes in our environment. Generally, the levels of organization may vary from a micro- to macrocontinuum that starts with organizational agents/individuals, components/subunits, a single entity/facility, and a multiunit of systems/collaborations/coalitions/networks/taskforces/consortiums [11, 12]. Typically, the level of complexity inherent in the public health challenge or crisis event determines the corresponding level of organizational complexity required in the response [7, 13]. Challenges or crisis events that are short-term or relatively minor may only require minimal, ad hoc, or temporary organizational responses. Within the modern healthcare environment, these can represent informal partnerships or formal structures appearing as short-term project teams or workgroups. More involved and long-term problems may require increasing levels of complexity within the organizational response. These long-term or complex organizational responses may be represented in the form of permanent departments or divisions within an organization, or they may even extend beyond organizational boundaries to include coalitions, collaborations, taskforces, and interagency network arrangements.

One common public health system problem-solving strategy used throughout the US and worldwide involves formulating networks of individuals and organizations to coordinate global-, national-, state-, regional-, county-, city-, or even community-level responses to health threats to individuals or populations. Such networks (e.g., coalitions, collaborations, consortiums, and taskforces) present opportunities to define common goals, shape strategy, achieve economies-of-scale through the sharing of resources and facilitate the centralized monitoring and measuring of progress toward stated objectives. However, one challenge for the public health informatics professional involves ensuring that the data, information, and knowledge needs of networks of stakeholders—ranging from patient advocates, health organizations, providers, community groups, public health departments, policy makers, and researchers—are all met with efficiency and effectiveness. The issues surrounding timely intelligence were on full display during the recent Ebola virus and Zika virus outbreaks.

Currently, there are no consistent measures or metrics to evaluate the efficiency and effectiveness of the ability of “smart” health networks—of any size or configuration—to leverage data, information, and knowledge to produce actionable intelligence from their efforts [3, 14]. In other words, there is no quantifiable set of* standardized* measures or standard operational definitions of what a smart or learning health network is now or what it should be in the future [15]. Within any public health knowledge environment, a wide variety of network structures can be assumed. The organization is viewed as a dynamic, complex, and adaptive entity whose size, structure, and other organizational determinants must be constantly evaluated to promote its ability to respond to internal and external challenges, threats, and opportunities that will impact individuals and/or the collective leveraging of actionable intelligence to ensure success in health system management [16–18].

### 2.2. Problem/Issue Complexity Factors Shaping Knowledge Environments

An analogy for problem/issue identification and response within any public health knowledge environment is the human immune response in which the human immune system assesses threats on a constant basis and determines if a foreign agent is a “friend” or “foe.” Once identified in a healthy immune system, the proper immune response is triggered. For a* friend* response, facilitation/proliferation strategies ensue, and, for a* foe* response, elimination/mitigation strategies ensue. Two critical components in the overall immune response system are the ability to retain a memory of this encounter and to demonstrate system learning to prepare for future encounters of a relatively similar nature. The same sort of dynamic occurs within a public health system network among its various organizational components, actors/agents, and events. Once a phenomenon (i.e., circumstance/event/activity/occurrence) is identified as a potential problem, either threatening or nonthreatening, system or collective memory is vetted for familiarity [18]. If sufficient memory of the phenomenon or something similar is found, the ideal response algorithm(s) (set of instructions) is/are identified, outlining the appropriate response mechanism. If no memory exists, a response must be determined on an ad hoc basis. Clinical or public health events/activities that allow favorable health outcomes (e.g., diffusion of best practices, strategic summits, introduction of new technology, disease screening and awareness campaigns, and new funding announcements) may be considered targets for facilitation/proliferation, whereas unfavorable events/activities (e.g., disease outbreaks, health or food recalls, medical errors, deviations from guideline concordant care, risk behaviors linked to disease spread, budget shortfalls, and staff layoffs) may be targets for elimination/mitigation. In either case, sufficient memory must be generated of the response algorithms (process/workflows, policies/procedures) that contributed to the event(s), pathways toward emergence, and/or remediation strategy to eliminate the threat. Learning in this context presents the ability to circumnavigate potentially harmful events that have the potential for reoccurrence or the ability to repeat/reinforce positive events that are beneficial [19]. Hence, the ability to extract actionable intelligence from stored memory is essential to overall public health system performance and an effective knowledge environment [3]. Two factors that shape this dynamic of event, memory evaluation, and learning within a knowledge environment are* familiarity* and* preparedness, *borrowed from the field of emergency preparedness [20].

**Figure 3:**
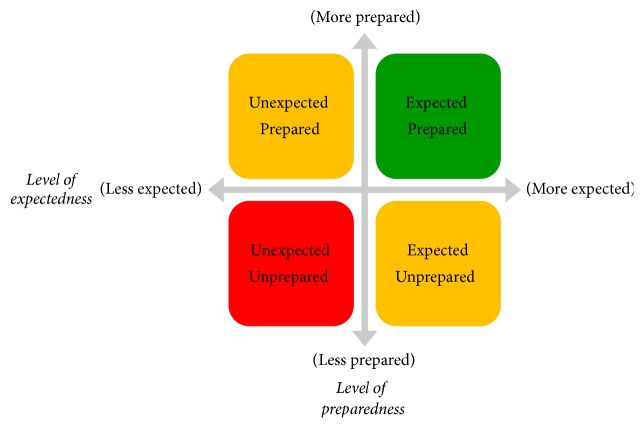
Problem/issue complexity factors.

Within any knowledge environment, issues/problem complexity and relative familiarity (stored memory) largely shape the level of “shock” or environmental stress to the public health system, which creates what Burton termed an organizational design misfit [16]. In the presence of an organizational design misfit, the goal is to seek to restore some measure of equilibrium [17]. The level of shock brought by the introduction of a problem/issue into any public health knowledge environment and its corresponding impact on the public health system can be thought of in terms of two factors: (1) the degree to which the event was expected to occur and (2) the degree to which the environment was prepared for its occurrence.  Figure 3 highlights the relationships of these two factors, where the green represents a highly desirable state of system and organizational readiness (operationally defined here as the agents'—within the public health knowledge environment—ability to process the event and determine an appropriate response), yellow represents less desirable states of organizational readiness, and red represents the least desirable state of organizational readiness and the highest level of vulnerability from both internal and external threats.

Although most public health systems are prepared to deal with any event; some noticeable changes can occur in the face of uncovered vulnerabilities introduced by shock events. Such adjustments on the organizational side may present as unexpected leadership shifts, sudden changes in organizational command structures, abrupt shifts in policy and procedures, new stratums of research funding to investigate and solve problems, or the addition or elimination of staff and key personnel [16]. On the public health knowledge environment side, such adjustments can take the form of wide-scale data integration or health information exchange efforts, the formation of new database solutions, the demand for new technology to monitor and track the problem, surveillance protocols, information systems, knowledge portals, decision-support systems, and changes in information resource-management protocols [12]. The level of complexity in both the problem/issue and the capability of the public health knowledge environment to process the event and mount an appropriate response heavily shapes the level of organization (or in some cases reorganization) required to mediate the threat or exploit the opportunity. Additionally, these changes—and more importantly the rate of changes in the organization in particular and the public health knowledge environment in general—may serve as proxy indicators for overall public health knowledge environment maturity in managing uncertainty. In other words, a health system or public health agency that has undergone frequent leadership changes, high staff turnover, frequent redrafting of strategic plans, and reorganizations in a relatively short span of time serves as a strong indicator of the lack of overall public health knowledge environment maturity [12]. Such a public health knowledge environment characteristically remains in a loop moving from crisis-to-solution to a new or remerging crisis-to-solution. In contrast, a mature public health knowledge environment will seek to identify and understand the patterns of organizational complexity and problem/issue complexity emergence and response. Properly stored, organized, and readily accessible system memory can greatly aid in achieving a more mature public health knowledge environment.

### 2.3. Situational Awareness Factors Shaping Information Environments

Previously, we stated that organizational complexity is shaped by external or internal factors in a given public health knowledge environment, requiring different levels of formal or informal organizational structures to manage their environmental challenges. We also mentioned that the level of complexity inherent in problems/issues and the corresponding system memory and preparedness will shape system-level responses to control and mitigate any perceived threats. Here, we formally define the term situational awareness (SA) as “the ability to make sense of an ambiguous situation. It is the process of creating [situational awareness] and understanding to support decision-making under uncertainty—an effort to understand connections among people, places, and events in order to anticipate their trajectories and act effectively” [21]. Endsley elaborated the definition for situational awareness (SA), stating that SA is comprised of three subdomains that shape individual understanding of some phenomena. These include (1) Situation Perception (defining the current public health condition), (2) Situation Comprehension (defining the relative public health threat or opportunity), and (3) Situation Projection (forecasting the public health outcomes of hypothesized trajectories) [22].

Within situational awareness, the two elements of organizational complexity and problem/issue complexity are combined and serve as contributing factors that determine the degree to which organizational structure and function are properly suited to facilitate unencumbered information processing. Previous organizational theories have described the organization, functioning within a given environment, as an information-processing entity (IPE) [16]. From this perspective, organizations are seen as sophisticated information processing and decision-making machines that act as if they have preprogrammed subroutines in managing the loop stages of information flow and organizational processes (model → input → transformation → output → feedback) [4]. Within the IPE view of an organization, we must understand information processing as a means of shaping organizational and individual decisions, behaviors, and communication patterns [18]. The flow of information and knowledge is codependent on our constant need to learn and share knowledge, largely shaping the structure of our social and organizational network arrangements [23]. Therefore, the need to know or cognitive demand of both individuals and organizations becomes a primary driver of IPE activity [18, 23]. Information processing for the sake of storing mountains of data, information, and knowledge resources as an end itself is meaningless in the context of efficiency, effectiveness, and viability in meeting public health system organizational missions, goals, and objectives. More precisely, the primary function of any level of IPE—from simple department units to complex multiorganizational networks or health information exchanges—is to respond to what agents/actors need to know, when they need to know it, and to support the choices/decisions that must be made as shareholders navigate through the health system, defined here as actionable knowledge or intelligence [3]. The public health organizational IPE will seek to leverage SA to maximize readiness to meet public health threats from the environment and to maximize public health knowledge environment agent/actor individual and/or collective intelligence in the performance of core public health tasks and functions. Therefore, public health-centric SA serves as a comprehensive measure of public health system smartness and is essential for any standardized assessment of public health system performance from a public health informatics perspective.

## 3. Smart Systems Vulnerability Index

**Figure 4:**
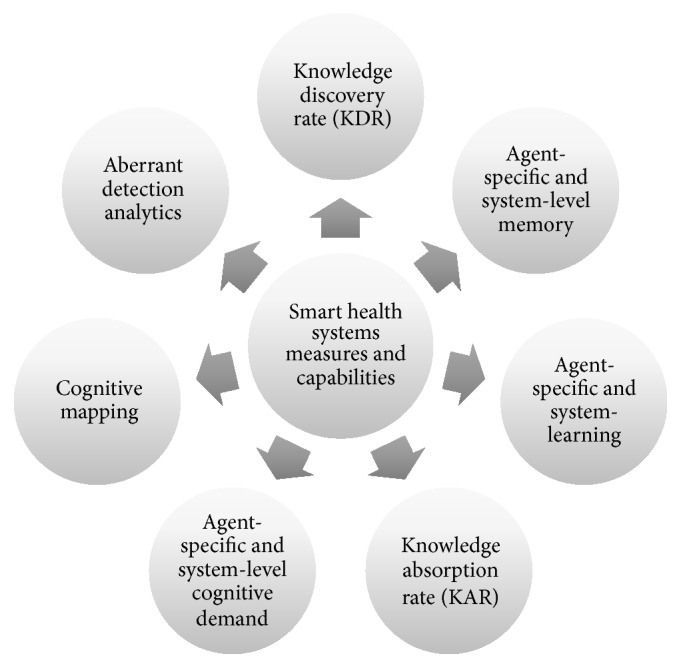
Smart health systems measures and capabilities.

In this section, we propose seven smart health system measures and capabilities appropriate for helping to manage public health organizational complexity, problem/issue/complexity, and situational awareness for public health systems networks and public health knowledge environments.  Figure 4 lists these seven measures and provides a brief description of a smart public health system and our rationale for its use. Although other measures may be available in the literature, we believe these seven effectively capture the key concepts discussed above. Of course, public health informatics professionals may need to use discretion when applying measures based on the context, the purpose of measurement, and any constraints hindering measurement.

### 3.1. Knowledge Discovery Rate (KDR)

Thomas Davenport described knowledge as data and information imbued with meaning and relevance. In this way, knowledge is seen as a continued aggregation and refinement that begins with raw data elements. For example, a set of 10 numerical digits constitutes raw and meaningless data. At the stage of information, it can be recognized as a telephone number.

These same digits can be viewed as knowledge when that number is contextualized as a conduit to satisfy some individual or organizational cognitive demand to support health decision-making or address some issue or problem. This telephone number can be viewed as a source of knowledge when, for example, it represents a nurse hotline for patient navigation. The informatics professional managing/building a mature public health knowledge environment works with stakeholders to develop a comprehensive knowledge inventory (formerly referred to as an ontology) of all products used to inform public health stakeholder decision-making [23].

The knowledge discovery rate (KDR) represents the rate at which knowledge is generated from new or existing data and information resources. In other words, the KDR shows how long it may take someone to (1) realize these 10 digits represent a telephone number, (2) process that this telephone number is connected to a patient navigating service, and (3) realize the nursing navigation service has additional resources to maximize the care experience. In public health, KDR may be expressed as the time it takes health officials to recognize a pattern in seemingly unrelated health events (e.g., ER traffic, school/work absence, provider case reporting, news reports, food plant inspection reports, and grocery store sales), indicating a disease threat in the form of a potential food-borne illness. It should be noted that display or interface is an essential component as well. Endsley explores how the interface and display of information can greatly deter the uptake of knowledge and consequently impair overall situational awareness [22]. For example, will everyone read these representations in the same manner—“1234567890” and “123.456.7890” and “(123) 456-7890”—in their communication exchange?

A key component in shaping the rate of knowledge discovery involves comprehensively assessing the presentation and display of data, information, and knowledge throughout key stages of any healthcare delivery (e.g., clinical pathway) or public health process. To that end, KDR involves understanding how knowledge is packaged for consumption in the form or paper or electronic tangible (explicit) knowledge products or as less tangible (tacit) knowledge products to inform decision-making. This measure examines the production curve of knowledge from generation, presentation, selection, and consumption, as well as the qualitative assessment of knowledge's relevance to agent-specific choice.

KDR may be particularly pertinent in settings in which pattern recognition depends on a coordination of data, information, and knowledge from a highly heterogeneous network of sources and stakeholders. KDR becomes essential in public health situations where intelligence has to be collated across multiple agencies (e.g., school, hospital, retail, and corporate), wide geographic boundaries (e.g., multiple regional metropolitan area health departments), or multiple categories of stakeholders (e.g., patients, providers, and health administrators). In such cases, KDR represents a measures of timeliness and operates as a key indicator of public health outcomes.

### 3.2. Organizational (Agent- or Systems-Level) Memory

Earlier we described the matrix of system shock as a function of preparedness and expectedness in response to internal and external stimuli (events). Organizational or systems-level memory can be described as the degree to which the history of these encounters, responses, and the relative degree of success or failure of those responses are catalogued and stored for future use by other agents/actors in the future. This can be operationally understood as the “repeatability” level, commonly referred to as level two of five in the capability maturity model (CMM) [24]. Systems memory simply asks to what degree are phenomena captured and labeled as favorable or unfavorable and response algorithms developed and made available for expedient consumption by the same and/or other agents within the knowledge environment. A lack of repeatability represents a high level of unnecessary “ad hoc” or CMM level-one responses [24] and may result in an inordinately high level of shock to the system for events that if properly catalogued could have been relegated to the realm of routine with minimal system-shock value.

Here, the primary measure is to determine the level of completeness, sophistication, and use of knowledge-bases that represent the sum of public health knowledge stored for current and future public health decision-making. This can be expressed as basic knowledge inventories, resource guides, policy and procedure manuals, and intranet/Internet lessons or best practices. It can also be expressed as highly sophisticated knowledge ontologies that capture and display public health knowledge, tasks, events, and procedures in complex electronic tools to support network modeling, information flows, and critical communication pathways. Public health knowledge portals can be constructed to identify public health stakeholder query demand more easily, as well as access, retrieve, display, and analyze knowledge use in any public health knowledge environment. This capability is essential to the proper use and maximization of organizational memory. In the absence of standardized knowledge memory management, a public health organization remains in a perpetual ad hoc response mode to each new or reoccurring public health crisis.

### 3.3. Agent-Specific and System Learning

There is a growing body of literature of the evolution of a “learning health system” [25]. Our study contributes to this concept by providing a conceptual definition of both agent-specific and system-level learning from the perspective of a public health informatics professional managing/building a public health knowledge environment. Here, learning is understood as the wisdom level of the informatics continuum [26]. We have refrained from using this concept throughout this discussion, but at this point it is appropriate to recognize that some informatics literature describes the informatics continuum (earlier referred to as the data progression) as data to information to knowledge to wisdom [26]. Typically, finding objective measures of wisdom is not easy or universally accepted. However, we have chosen to substitute wisdom for decision and outcomes. As a result, our data progression extends to the following sequence: data to information to knowledge to decisions to outcomes. The difference is that choices, when properly linked to specific outcomes and their corresponding consequences, provide opportunities for learning. As a result of this substitution, we are now able to define the concept of wisdom operationally as the degree to which choice—informed by relevant knowledge products—can lead to more highly desirable decisions, beneficial outcomes, and positive consequences for the overall health and wellbeing of agents and the system.

We extend our definition of wisdom to incorporate intelligence, simply understood as the display of wisdom over time. In our model, learning acts as a measure of differential wisdom and intelligence over time (the difference measured at two distinct points in time). In other words, this equation involves individual or organizational wisdom displayed or measured at some endpoint (*t*2) minus the individual or organizational wisdom displayed at some starting point (*t*1). The organizational IQ in a learning health system is then understood as the measure of differential wisdom displayed over time toward some set of decisions/choices, actions/tasks, or other health phenomena. Learning represents a measurement of agent-specific or system-level discernment (the ability to leverage situational awareness in comprehending threat level, as well as leveraging stored or new knowledge in choosing between differing options). To this end, learning is construed as the means of refinement in the art of discernment or wisdom acquisition.

In public health terms, the operational construct of this measure of learning is still evolving, and little literature exists on applying this construct in public health practice. We suggest that measures of learning—presented here as a means of leveraging knowledge resources in a wise manner—are largely dependent on the previous measures of organizational memory. In the absence of a well-designed public health knowledge-base that captures history or practice, learning from such experience becomes extremely episodic and anecdotal in nature. For example, we can only speculate on how much stored memory has been gathered with respect to the Ebola crisis that may more easily mitigate another outbreak or similar outbreaks of other diseases in related conditions. The emergence of rapid learning health networks deals with some aspects of this challenge by streamlining the processing of research evidence into practice and gathering knowledge stores of what works best in achieving better health outcomes. However, global implementations of these research-to-practice and comparative effectiveness networks are still in the early stages of development.

### 3.4. Knowledge Absorption Rate (KAR)

Carley et al. described how the sum of knowledge within a given system boundary can be quantified in terms of knowledge bits [27–29]. According to this concept, knowledge represented in its various forms can be deconstructed into quantifiable units [29, 30]. The number of knowledge units or bits that may comprise a discreet package of knowledge is determined by the level of complexity of the decisions or tasks this knowledge is designed to inform [29, 30]. As such, a direct relationship exists between the number of knowledge bits and the level of the complexity in related decisions and tasks. The greater the level of task or decision complexity/criticality or decision, the larger the knowledge complement (or number of knowledge bits) associated with the management, storage, display/representation, diffusion, use, and comprehension of knowledge [29, 30]. This perspective assumes more knowledge bits are needed to saturate or carry out a complex task or make a critical choice than to implement a more simplified/less complex task or choice.

In essence, this concept of knowledge bits suggests that throughout the knowledge environment, agents/actors can have either 0% saturation of knowledge (or 0 bits) up to 100% saturation of knowledge (or all bits available in the knowledge environment). The consumption rate or absorption of these knowledge bits over time, in the performance of core task performance, can then be evaluated using a variety of statistical and computational modeling methods. To carry this out, a value or weight is assigned to every piece of knowledge represented within a knowledge inventory (also referred to as ontology). The weight given to a knowledge product represents both the degree of value assigned by consumers of that knowledge product (elasticity-of-demand) and the magnitude of importance of the respective decision(s) it is intended to inform (criticality). The curve of a knowledge product's elasticity-of-demand and criticality of decisions is evaluated in the context of a core set of tasks to be performed at the agent or system level.

In public health settings, KAR represents a concrete way of measuring overall application of knowledge to performance. In previous studies, we examined the knowledge absorption rate of community health clinical staff with regard to breast, cervical, and colorectal cancer screening policies, guidelines, and protocols as derived from the use of electronic clinical decision-support (CDS) [31]. We examined the extent to which CDS use and corresponding knowledge absorption rates would be correlated to organizational performance for cancer screening [31]. We demonstrated that KAR was, in fact, a predictor of organizational performance in meeting process-of-care outcomes in cancer care [31]. Hence, we suggest that KAR can serve as an effective measure of HIT impact on performance by focusing on end-users' ability to access key knowledge by interacting with HIT tools and applying this knowledge to healthcare delivery and public health practice.

### 3.5. Agent-Specific and System-Level Cognitive Demand

Within any given knowledge environment, agents/actors at many levels perform key tasks, make decisions, and engage in a series of activities that can be described by a set of process algorithms [28]. The constant factor governing this activity is the principle of a cognitive demand for information. The principle of supply and demand borrowed from the field of economics applies somewhat to the field of informatics with respect to agents'/actors' need for information to support decision-making and task performance. Here, we focus on the metric as a measure of the relative demand for data, information, and knowledge resources by agents/actors operating at all levels of the multilevel model, as well as the corresponding supply of data, information, and knowledge resources available for consumption. We refer to main driver of the interplay between the supply and demand of data, information, and knowledge resources as the cognitive demand or simply the “need to know.”

This “need to know” or cognitive demand shapes information-seeking behaviors of the agents/actors within the system and may govern the amount effort they are willing to expend in acquiring the data, information, or knowledge resources. The level of importance or criticality of information to the agent is measured by the elasticity-of-demand (a borrowed term) for that information. The measure of elasticity coupled with the relative supply of information can be used to measure relative states of “informedness” of the agents/actors within the system. According to the formal definition of elasticity, in an elastic demand, the change in quantity demanded due to a change in price is large [32]. In contrast, an inelastic demand is one in which the change in quantity demanded due to a change in price is small [32]. Cognitive demand can serve as a core measure in identifying knowledge-related vulnerabilities within a system or the relative degree to which the cost of the knowledge required is acceptable or not acceptable.

**Figure 5:**
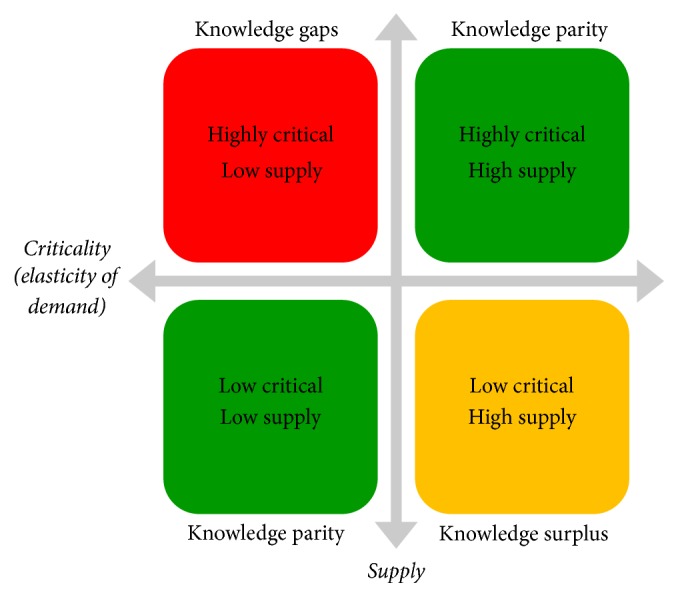
Criticality (elasticity-of-demand) and the supply of knowledge.

We understand that in the context of health systems, the concept of price with respect to knowledge can be measured in terms of access, affordability (time and effort), overall opportunity-cost (ease-of-use, processing, comprehension, and understanding), and relevancy.  Figure 5 lists that four distinct states agents/actors can assume within any public health knowledge environment based on the level of criticality (elasticity-of-demand) and the supply of knowledge. When the cognitive demand for knowledge is highly critical and the relative supply is limited, knowledge gaps emerge. Such knowledge gaps may result from a variety of scenarios, including (1) the information or knowledge product does not exist, resulting in a need for innovation; (2) the resource exists, but access is in some way limited or encumbered; (3) the resource exists with abundant access but is not easily processed or consumed because of literacy challenges, content presentation, or other reasons; and (4) the supply is challenged by other competing priorities and is intentionally undeveloped or underdeveloped. The two states we refer to as parity conditions represent areas where the level of criticality is adequately met by the level of knowledge supply. In such cases, the main strategy is to employ continuous monitoring to ensure balances remain within desired ranges of acceptability in conjunction with the need for balance in the overall public knowledge environment. A state of knowledge surplus results when the level of information or knowledge product supply exceeds the relative level of importance placed on the information or knowledge products (also understood as relevancy). This state represents an opportunity for the elimination of outmoded or underused knowledge resources, information system redesign/upgrade, or other information technology strategic efforts to ensure long-term relevance of information resources, information systems, and knowledge products.

The public health application of this measure of relative cognitive demand or the need to know is illustrated by the recent Zika outbreak. It became evident that the population at greatest risk for the disease was pregnant or soon-to-be pregnant women. The demand for information regarding protective measures, travel restrictions, the rate of transmission, the relative threat to fetal health, and signs and symptoms once infected created large pockets of health consumer uncertainty, stress, and anxiety. Given the severe level of risk to pregnant women and their developing babies, the demand for knowledge of what to do for protection was highly critical. The window of transmission of the Zika virus from mother to fetus was highly uncertain, the effectiveness of preventive measures was hard to measure, and travel decisions to affected areas were rather unclear, resulting in conditions of highly critical and low-resourced knowledge profiles for many health consumers and stakeholders at all levels. Rapid research was needed to identify proven measures against the invading mosquito population. Public health departments throughout the affected areas were scrambling to model the spread of the disease, measure the impact of the preventive measures, and manage the reports of news cases. Meanwhile, the public was constantly demanding new answers and updates on a daily basis. This was compounded by the timing of the 2016 Summer Olympic games in Rio that sparked highly publicized athletes refusing to travel to the region to participate in the event. Highly critical/low supply-resourced conditions are probably the most difficult to manage. In any public health knowledge environment, a continual assessment of stakeholder cognitive demand must be done—relative to the capabilities of the existing or evolving knowledge-base—as a means of satisfying current and/or projecting anticipated demands.

### 3.6. Cognitive Mapping

Once the knowledge inventory and relative measures of importance are assessed and the corresponding process and information flows have been identified, the public health informatics professional can now engage in the process of creating cognitive maps or models of both existing and emerging knowledge and communication pathways. These pathways can be modeled for specific agents, for the system as a whole, or any combination of the two. Here, the public health informatics professional is not simply asking who uses what information or examining the use of computerized information resources; instead, the goal involves trying to model the cycle of information and knowledge within the public health knowledge environment. This information cycle is best understood as starting with raw materials, in this case raw and at times unformatted data elements, which are assembled into chunks of information (e.g., electronic databases or information systems). These information chunks are either coordinated in the formation of meaningful knowledge products or presented to users of information to coordinate based on their specific needs (structured queries), which can be thought of as off-the-shelf knowledge products or ad hoc user-defined knowledge products to support decision-making (ad hoc queries). We refer to this cycle as knowledge refinery.

Analytic measures of knowledge refinement consist of examining the pace of knowledge development and exploring system responsiveness as expressed by the supply of and demand for data, information, and knowledge resources [33]. The basic elements of analysis consist of the total knowledge in any given public health knowledge environment (knowledge entropy) relative to the amounts of used kinetic knowledge and unused potential knowledge [33]. This can also be expressed in terms of the amount of knowledge/information gained or loss in an effort to maximize performance [33]. Additionally, the public health informatics professional could examine existing and emerging pathways that are developed through the examination of patterns of use, which is closely linked to the concept of plasticity [34]. In the field of neuroscience, the term neuroplasticity refers to the human brain's ability to change in response to behavioral, environmental, and neural processes [35]. In the human brain, these pathways, after repeated stimulation and reinforcement, are actually carved into the brain tissue [35]. Like neural pathways are carved into the human brain, IPEs, as described earlier, may examine how public health knowledge environments respond to changes in behavior, environmental conditions, or agent-specific or system-level cognitive demands [34, 36].

Pathways of changes in public health knowledge environment cognition can be modeled using a variety of conceptual and visualization techniques [37]. Such pathways, when observed and modeled, can yield repeated patterns, which may be canonized as permanent or semipermanent cognitive pathways toward system-level knowledge and learning health systems [37]. Within our model of public health knowledge environments, highly intelligent health systems have the ability to manage such cognitive pathways in response to cognitive demands [37]. Where old or unused pathways exist, data and information systems (and the corresponding knowledge products) will likely be considered outdated or not useful. Where current cognitive pathways are robust and frequented, data and information systems are likely to be considered essential to decision-making, and where new and emerging cognitive pathways are observed or predicted, the likelihood exists for innovation and systems development to support emerging communities of practice, workgroups, department/divisions, and other formal or informal organizational structures [38].

The public health application lies in understanding the public health system as an evolving complex network of individuals, organizations, groups, and knowledge resources. Here, the public health informatics professional may find knowledge, skills, and abilities in modeling social networks and organizational networks that are essential in establishing current state network diagrams (baseline) and future state diagrams designed to guide the visualization of a public health knowledge environment. In this context, a large library of network measures can be employed to support the analysis of a public health system and its respective knowledge environment, like measures of network density agents, closeness/connectedness of agents to each other or to other knowledge resources, patterns of clustering and cliques behaviors, knowledge-sharing practices, and more. The application of social and organizational network analysis in public health is growing at a rapid rate and is expected to continue moving forward.

### 3.7. Aberrant Detection Analytics

Arguably the most important analysis within this discussion involves being able to detect subtle changes within the public health knowledge environment that may pose a threat to one or more agents or the system overall. Here, we discuss the ability of intelligent analytics or telemetry as part of a* Public Health Situation Room* that can be used to detect subtle changes within the public health knowledge environment. The public health informatics professional relies heavily on the use of probes and sensors as part of any surveillance and monitoring system to gather intelligence. Similarly, physicians and nurses rely on telemetry to monitor patient vital signs, drivers use dashboards to detect changes in automobile status, and investors use tickers to track global investments. These forms of monitoring and tracking systems have one feature in common: they all make use of a grid system or network of core indicators as validated predictors of overall system health.

Any sensor network used to monitor and track activity within a given public health* Public Health Situation Room* must recognize several information-specific concepts. First, information does not always travel along predefined organizational departmental or process pathways. Instead, information exchange may occur along a multiplicity of pathways, some predictable and others highly unpredictable. While organizational constructs of departments and divisions may account for some of communication and information exchange, they do not account for all activity. Therefore, the placement of public health data/event collecting sensors within a given public health knowledge environment must be a fluid network that is highly adaptive and capable of capturing activity in different settings, wherever the information channels may lead.

Second, the* Public Health Situation Room* sensor grid must be able to identify and track activity by both internal agents and system components, as well as by external agents and system components that may interact with the environment. No public health knowledge environment is completely closed. As a result, sensors must capture intelligence from portals through which information travels in and out of the system in all forms. Finally, the level of completeness must be defined to determine what represents an adequate level of coverage. A poorly designed or partial* Public Health Situation Room* sensor grid that allows large levels of undetected activity would not be useful on long-term. A sensor grid should be viewed as a living and growing network of data/event collection activities that changes with evolving needs and priorities, and the level of granularity or specificity of detection must also be capable of changing within the grid as strategic priorities shift. The full list of indicators drawn from public health knowledge environment factors, stakeholder-levels, and unique views will largely shape the types of sensors, density of the network, and level of sensitivity needed for meaningful aberrant detection algorithms and monitoring system development.

Public Health Situation Rooms are used at all levels of the public health system throughout national and international health settings, the US Department of Health and Human Services, various public health agencies, and healthcare delivery settings across the globe. However, there is no standardized model for this type of monitoring capability. Public Health Situation Room needs and priorities vary widely by organization and may include but not be limited to disease management, outbreaks investigation, emergency preparedness, disaster response, community health assessments, and even healthcare access, equity, and quality. The use of electronic performance, strategic, operational, and clinical dashboards are typical of such Public Health Situation Rooms. We argue the primary challenge of the public health informatics professional in the design and execution of Public Health Situation Rooms is to develop the underlying smart infrastructure (knowledge-base) and array of analytic measures described in this discussion to ensure the maximum impact on desired outcomes.

## 4. Conclusion

As we enter a public health informatics era with terms like learning health systems, smart health systems, and adaptive complex health systems, we must identify a common set of analytic measures and capabilities to inform our modeling, measuring, and managing of public health “smartness.” Such a set of measures must take into account the full spectrum of sociotechnical factors that make up a public health system and shape performance, including technical, organizational, and human contributions. It is essential that we understand the basic drivers of smart systems, expressed in this discussion as simply the need to know or cognitive demand. This basic need to know and our corresponding effort to leverage data, information, and knowledge resources toward some individual or collective set of goals and objectives form the basic parameters of any smart system. In the context of a public health system, public health informatics professionals stand poised to redefine the benefit of smarter healthcare delivery and public health practice. A common set of analytic measures and capabilities that can drive efficiency and viable models can demonstrate how incremental changes in smartness generate corresponding changes in public health performance. Here, we introduced the concepts of organizational complexity, problem/issue complexity, and situational awareness as three codependent drivers of smart public health systems characteristics. We also propose seven smart health systems measures and capabilities that are considered essential in a public health informatics professional's toolkit. Because this area of research and practice is still in its formative stages, the intent of this discussion is to build on the developing body of literature seeking to establish standardized measures for smart, learning, and adaptive public health systems.
